# Supplementary material for: Higher Diet Quality in Latina Women during Pregnancy May Be Associated with Sociodemographic Factors
Source: Int J Environ Res Public Health. 2022 Oct 26;19(21):13895. doi: 10.3390/ijerph192113895 (PMC9657950; doi:10.3390/ijerph192113895)
Supplement: Supplementary file 1 [file ijerph-19-13895-s001.zip › ijerph-1882354-supplementary.pdf]

Supplement Table S1. Average diet quality score differences by acculturation and nutrition literacy levels.

Diet quality components	Maximum points	Overall (n=99)	Low AC and Low NL [1] (n=59)	Low AC and Moderate NL [1] (n= 15)	Bicultural/high AC and Low NL [1] (n=17)	Bicultural/high AC and Moderate NL [1] (n=8)
		Mean Score (95%CI)				
Total HEI-2015	100	69.8 (67.8, 71.8)	70.0 (67.1, 73.0)	67.8 (62.5, 73.1)	65.8 (59.0, 72.6)	66.1 (55.2, 77.1)
Total fruits	5	3.5 (3.2, 3.8)	3.7 (3.2, 4.1)	3.7 (2.8, 4.5)	3.6 (2.6, 4.7)	2.9 (1.2, 4.6)
Whole fruits	5	3.9 (3.6, 4.2)	4.7 (4.3, 5.0)	4.7 (4.0, 5.0)	4.7 (3.9, 5.0)	4.0 (2.7, 5.0)
Total vegetables	5	3.7 (3.4, 4.0)	3.8 (3.4, 4.2)	3.8 (3.0, 4.5)	3.7 (2.8, 4.7)	2.9 (1.4, 4.3)
Greens and beans	5	3.6 (3.3, 4.0)	3.8 (3.3, 4.3)	4.1 (3.2, 5.0)	3.6 (2.4, 4.8)	3.0 (1.1, 5.0)
Whole grains	10	7.6 (6.9, 8.4)	7.1 (6.1, 7.9)	6.3 (4.6, 7.9)	5.8 (3.7, 7.9)	7.7 (4.4, 10.0)
Dairy	10	6.6 (6.1, 7.1)	6.3 (5.6, 7.1)	7.2 (5.8, 8.5)	6.4 (4.6, 8.1)	7.4 (4.6, 10.0)
Total protein foods	5	4.7 (4.5, 4.8)	4.7 (4.5, 4.8)	4.9 (4.5, 5.0)	4.4 (3.9, 4.8)	4.7 (4.0, 5.0)
Seafood and plant proteins	5	3.8 (3.5, 4.2)	3.8 (3.2, 4.3)	4.3 (3.4, 5.0)	3.7 (2.5, 5.0)	4.0 (2.0, 5.0)
Fatty acids	10	4.7 (4.1, 5.4)	5.0 (4.8, 5.1)	3.5 (2.8, 4.2)	4.3 (3.4, 5.2)	5.1 (4.3, 5.9)
Refined grains	10	7.5 (6.9, 8.1)	7.2 (6.3, 8.1)	7.5 (5.9, 9.0)	6.7 (4.7, 8.7)	6.4 (3.2, 9.6)
Sodium	10	4.6 (4.0, 5.3)	5.1 (4.1, 6.0)	4.9 (3.2, 6.6)	4.0 (1.8, 6.2)	5.8 (2.3, 9.3)
Added sugars	10	8.1 (7.6, 8.5)	8.2 (7.5, 8.8)	7.2 (6.0, 8.4)	7.2 (5.6, 8.7)	8.4 (6.0, 10.0)
Saturated fats	10	6.9 (6.3, 7.4)	7.2 (6.4, 7.9)	6.0 (4.6, 7.3)	7.9 (6.1, 9.6)	5.9 (3.0, 8.7)

Abbreviations: AC, acculturation; NL, nutrition literacy; CI, confidence interval; HEI-2015, The Healthy Eating Index-2015; Notes: The HEI-2015 is a measure of diet quality used to assess how well a set of foods aligns with the 2015-2020 Dietary Guidelines for Americans. The HEI-2015 includes 13 components that can be summed to a maximum total score of 100 points. A higher score indicates a diet that aligns with the Dietary Guidelines. [1] Adjusted for age, nativity, time living in the US and education attainment.
